# Supplementary material for: Potential Treatment of Lysosomal Storage Disease through Modulation of the Mitochondrial—Lysosomal Axis
Source: Cells. 2021 Feb 17;10(2):420. doi: 10.3390/cells10020420 (PMC7921977; doi:10.3390/cells10020420)
Supplement: Supplementary file 1 [file cells-10-00420-s001.pdf]

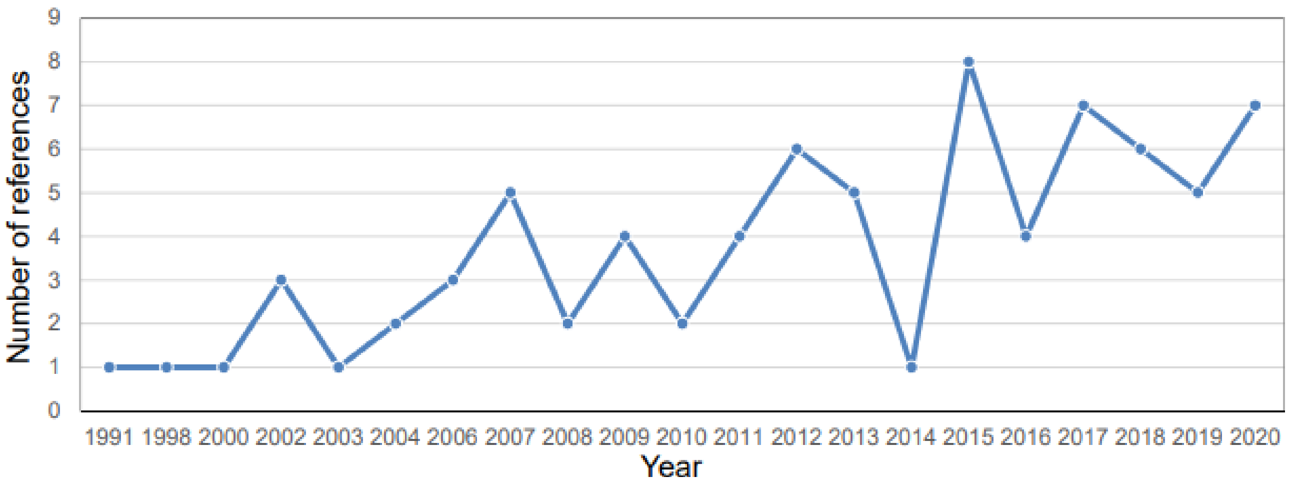


**Figure S1.** Information on the year of publication of research papers referenced in this review. 47% of the references have been published since 2015, indicating that this review article is up to date.
